# Rheumatic Heart Disease and Myxomatous Degeneration: Differences and Similarities of Valve Damage Resulting from Autoimmune Reactions and Matrix Disorganization

**DOI:** 10.1371/journal.pone.0170191

**Published:** 2017-01-25

**Authors:** Carlo de Oliveira Martins, Lea Demarchi, Frederico Moraes Ferreira, Pablo Maria Alberto Pomerantzeff, Carlos Brandao, Roney Orismar Sampaio, Guilherme Sobreira Spina, Jorge Kalil, Edecio Cunha-Neto, Luiza Guilherme

**Affiliations:** 1 Heart Institute (InCor), School of Medicine, University of São Paulo, São Paulo, São Paulo, Brazil; 2 Institute of Investigation in Immunology, National Institute for Science and Technology, University of São Paulo, São Paulo, São Paulo, Brazil; Cincinnati Children's Hospital Medical Center, UNITED STATES

## Abstract

Autoimmune inflammatory reactions leading to rheumatic fever (RF) and rheumatic heart disease (RHD) result from untreated *Streptococcus pyogenes* throat infections in individuals who exhibit genetic susceptibility. Immune effector mechanisms have been described that lead to heart tissue damage culminating in mitral and aortic valve dysfunctions. In myxomatous valve degeneration (MXD), the mitral valve is also damaged due to non-inflammatory mechanisms. Both diseases are characterized by structural valve disarray and a previous proteomic analysis of them has disclosed a distinct profile of matrix/structural proteins differentially expressed. Given their relevance in organizing valve tissue, we quantitatively evaluated the expression of vimentin, collagen VI, lumican, and vitronectin as well as performed immunohistochemical analysis of their distribution in valve tissue lesions of patients in both diseases. We identified abundant expression of two isoforms of vimentin (45 kDa, 42 kDa) with reduced expression of the full-size protein (54 kDa) in RHD valves. We also found increased vitronectin expression, reduced collagen VI expression and similar lumican expression between RHD and MXD valves. Immunohistochemical analysis indicated disrupted patterns of these proteins in myxomatous degeneration valves and disorganized distribution in rheumatic heart disease valves that correlated with clinical manifestations such as valve regurgitation or stenosis. Confocal microscopy analysis revealed a diverse pattern of distribution of collagen VI and lumican into RHD and MXD valves. Altogether, these results demonstrated distinct patterns of altered valve expression and tissue distribution/organization of structural/matrix proteins that play important pathophysiological roles in both valve diseases.

## Introduction

Rheumatic fever (RF) results after approximately two weeks of a throat infection by *Streptococcus pyogenes* and affects non-treated susceptible children and teenagers. Rheumatic heart disease (RHD) is the major sequel occurring in approximately 60% of the cases. Recently, an update of the disease noted that greater than 34 million cases of RHD are estimated to occur worldwide, causing over 10 million disability adjusted life years and 275,000 deaths each year (reviewed by Carapetis *et al*. and by Remenyi *et al*.) [1,2].

The disease is genetically controlled, and several genes implicated in the development of humoral and Th1 (interferon-gamma and TNF-alpha) cellular immune responses that lead to the development of RF/RHD autoimmune inflammatory lesions are currently better understood (reviewed by Guilherme*et al*.) [3].

Rheumatic fever manifests initially as polyarthritis followed by cardiac manifestations in 30 to 40% of children. The condition affects the myocardium and mitral and/or aortic valves, leading to permanent valve damage and RHD.

Myxomatous degeneration (MXD) is another valvulopathy that preferentially affects mitral valve and is characterized by mucopolysaccharide accumulation into the valvular tissue with fragmentation and disruption of the elastic fibers and collagen bundles, leading to valve prolapse and even to chordae rupture [4,5]. This disease is not associated with inflammation in contrast to RF/RHD and is the most common valve disease in the United States and Europe [6]. A descriptive large-scale proteomic study on mitral valves of RHD and MXD patients highlighted different key proteins associated with the development of valve lesions in these conditions. Briefly, structural and extracellular matrix proteins exhibited altered expression in both diseases. Specifically, vimentin, lumican and vitronectin exhibit increased expression, whereas biglycan and collagen VI exhibit reduced expression in valves of RHD patients as compared to control valves. In contrast, all these proteins displayed reduced expression in MXD valves [7].

In the present study, we used immunoblot analysis to confirm previous large-scale proteomic analysis [7], as well as immunohistochemistry and confocal microscopy to assess whether tissue distribution of vimentin, collagen VI, lumican, and vitronectin were also altered in valve-tissue lesions of both RHD and MXD patients as compared to control valve tissue.

## Material and Methods

### Study Design

Chronic RHD results from organization of acute and recurrent episodes of acute rheumatic fever and mitral valve is the most affected. Post inflammatory scarring leads to chronic fibrotic valvular deformities. RHD histological valvular features are disorganization of the original layered architecture of the cusps with marked fibrosis, thick-walled muscular vessels proliferation and focal lymphocytic inflammatory infiltration, with or without calcification. In MXD, microscopically, there is an excessive accumulation of proteoglycan material distributed within the cusp spongiosa layer with degeneration and loss of collagen and elastic tissue.

Clinical and histological criteria were used for diagnosis of both RHD and MXD patients. All patients underwent surgical procedure as they presented severe valvular dysfunction (Table 1). Fragments from the mitral anterior leaflet were obtained at the time of valve surgery, frozen and stored in liquid nitrogen. The histological processing was performed at maximum of 6 months after surgical collection. Samples were fixed in 10% neutral buffered formalin for immunohistochemistry and in acetone for confocal microscopy. The histological data were evaluated by two experienced pathologists from Heart Institute, School of Medicine, University of São Paulo.

**Table 1:** Clinical data and mitral histological findings.

Patients	Age	Gender	Clinical data and mitral histological findings
**RHD**			
1	57	F	Regurgitation, chronic valvulitis and fibrosis.
2	21	M	Regurgitation, acute valvulitis, verrucae and Aschoff bodies, vascular neoformation, fibrosis and calcification.
3	25	F	Regurgitation, diffuse fibrosis, vascular neoformation and focal calcification.
4	51	F	Stenosis, chronic valvulitis, fibrosis, vascular neoformation, calcification and scarce lymphomononuclear infiltrate.
5	64	F	Regurgitation, chronic valvulitis and fibrosis.
6	38	M	Stenosis, chronic valvulitis, fibrosis, vascular neoformation, calcification and scarce lymphomononuclear infiltrate.
7	37	F	Stenosis, chronic valvulitis, fibrosis, vascular neoformation, calcification and moderate lymphomononuclear infiltrate.
8	56	M	Stenosis, chronic valvulitis, vascular neoformation, fibrosis, calcification and mild lymphomononuclear infiltrate.
**MXD**			
9	39	M	Diffuse myxomatous alterations, fibrosis and mild lymphomononuclear infiltrate.
10	75	M	Focal myxomatous alterations, difuse fibrosis, mild lymphomononuclear infiltrate and calcification.
11	73	M	Diffuse myxomatous alterations and fibrosis.
12	62	M	Diffuse myxomatous alterations and fibrosis.
13	86	M	Diffuse myxomatous alterations, fibrosis and mild calcification.
14	53	F	Diffuse myxomatous alterations and discrete fibrosis.
15	73	F	Diffuse myxomatous alterations and focal fibrosis.
16	61	M	Focal myxomatous alterations and diffuse fibrosis.

Mitral valve fragments from RHD patients (n = 8; 3 male and 5 female; mean age 44 years) and MXD patients (n = 8; 6male and 2female; mean age 65 years) were obtained at the time of valve surgery. Mitral valve fragments from cadaveric organ donors without a history of valvulopathy were used as a control group (CTL) (n = 6; 3male and and3 female; mean age 43 years). The histological findings of patients included in this study along with their clinical data are presented in Table 1.

This study was approved by the Ethics Committee from Heart Institute, School of Medicine, University of São Paulo (number 0053/07). Signed informed consent was obtained from all patients or their relatives prior to the inclusion in this study.

### Immunoblotting

Protein extracts were separated by one dimension polyacrylamide gel electrophoresis 12% (SDS-PAGE) with molecular weight markers (S1 Fig) in a side lane, transferred to nitrocellulose membranes for 1 hour and 30 minutes and blocked with skim milk in TBS-Tween for one hour. For collagen-VI detection, the proteins were transferred in a wet-system overnight at 4°C. The proteins were probed against specific antibodies to vimentin (ab45939, 1:2000, Abcam, San Francisco, CA), lumican(sc-166871, 1:2000, Santa Cruz, Heidelberg, Germany), collagen-VI alpha chain (sc-20649, 1:2000, Santa Cruz, Heidelberg, Germany), and vitronectin (sc-7776, 1:1000, Santa Cruz, Heidelberg, Germany).The expression level of beta-actin (ab8226, 1:5000, Abcam, San Francisco, CA, USA) was used to control for equal loading.

Densitometry analysis of immunoblots of all proteins mentioned above was performed using the Image Quant software program purchased from GE Healthcare, Upsalla, Sweden. Relative protein expression was calculated as pixel volume in specific band/pixel volume in beta-actin band for each lane.

### Vimentin isoform identification

Vimentin spots of different molecular weight were previously identified by mass spectrometry in our RHD valvar proteomic study [7]. Briefly, spots were excised, reduced, alkylated with iodoacetamide, and submitted to controlled proteolysis. The mass-to-charge ratios of the proteolysis-derived products were measured by ESI-MS/MS (Ultima, Waters™). The quantified ionic masses were compared to primary sequences in the Swiss-Prot human database using the MASCOT search algorithm [8].

**Fig 1:**
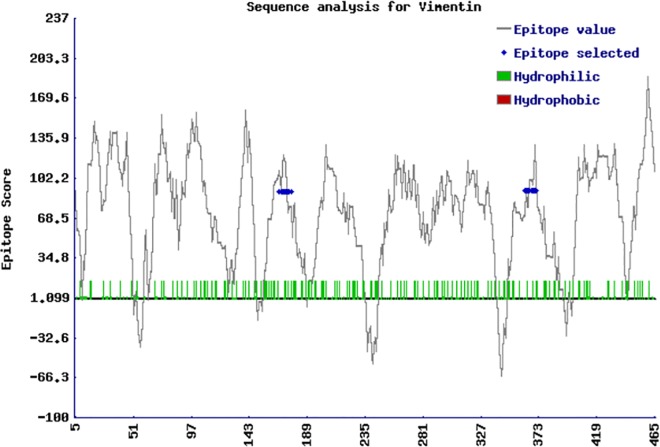
Selected sequences in the N- and C-terminal regions of vimentin for eliciting anti-vimentin rabbit polyclonal sera. Mass spectrometry-identified peptides from the N- and C-terminal fragments of vimentin were used to induce rabbit polyclonal antisera which were used in subsequent experiments. Antisera were raised by AB-mart Inc. Epitope score plot was performed with a proprietary algorithm from AB-Mart Inc.

To identify the specific reactivity against two immunogenic 15-mers of both N-terminal (163-177-QLTNDKARVEVERDN) and C-terminal (358-372- YQDTIGRLQDEIQNM) fragments of vimentin identified by the MASCOT data base, as mentioned above, two rabbit specific polyclonal antibodies were manufactured by AbMART-Shanghai Co. LTD as per our specific request (project 18741–1, contract No2014 040211990, 1:500 dilution) (Fig 1).

### Immunohistochemistry and confocal microscopy

Serial 6-μm sections of frozen fragments from anterior leaflet of all patients were transferred to silane-treated microscope slides for *in situ* evaluation of vimentin, collagen-VI and lumican localization.

For immunohistochemistry analysis, valve slices were fixed in 10% neutral buffered formalin and probed for vimentin (1:500), lumican (1:200) and collagen-VI alpha chain (1:500). A secondary step was performed using secondary IgG antibody (ABC kit VECTASTIN, Vector Laboratories, USA). Reactions were developed with diaminobenzadine (DBA) (Sigma). Histological and immunohistochemistry analysis and documentation were performed using manual microscope Axioskop 2 Plus (Zeiss, Germany) equipped with AxioCam HR color camera and Axiovision Release 4.8.2 SP2 software.

Confocal microscopy was also performed to evaluate the co-localization of collagen-VI and lumican in valve samples of RHD and MXD patients (# 2, 5, 6, and 8) and MXD patients (#10, 14, 15 and 16) and valve sample of two cadaveric donors as controls. Valve slices were fixed in acetone, washed five times with phosphate buffer solution (PBS) and submitted to treatment with 40 μl PBS-Triton 0.2% for 30 minutes, followed by the addition of 40 μl of 2% PBS-BSA (buffer solution with bovine serum albumin). Samples were incubated in anti-collagen VI alpha chain (1:100 dilution) and anti-lumican antibodies (1:50 dilution) for 15 hours at 4° C under humidity. Then slides were washed 5 times in 0.5% PBS/Tween, and 40 μl of secondary anti-collagen VI and anti-lumican (rabbit 1:200) antibodies in PBS-BSA-DAPI (4,6 diamidine-2-pheylindole hydrochloride) (Roche) were added. The slides were incubated in a humidity chamber for 45 minutes at room temperature in the dark and washed three times in PBS/Tween for 3 minutes each. The tissue-images were observed under oil immersion (63X objective) on an inverted laser confocal microscope (Zeiss LSM510-Meta) at the Confocal “Rede Premium” Multiuser Facility from Heart Institute, University of São Paulo.

### Statistical analysis

Comparisons of protein expression between the control and diseased groups were performed with the ANOVA (Kruskal -Wallis) *t-test*.

## Results

### Vimentin N and C-terminal portions as target of autoimmune reactions

**Fig 2:**
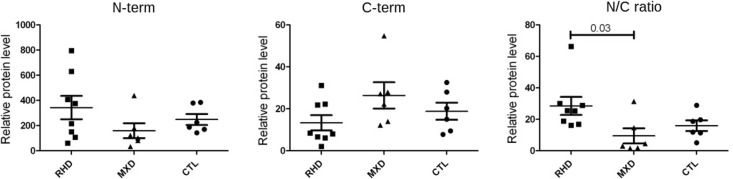
Relative expression of N-terminal and C-terminal portions of vimentin in valves of RHD and MXD patients. Immunoblots of valve proteins in SDS-PAGE gels were made with antisera raised against vimentin N-terminal or C-terminal peptides. Relative protein expression levels were measured by densitometry. RHD valve samples (n = 8), MXD valve samples (n = 8) and controls (n = 6). Vimentin N-term and C-term: N and C terminal fragments; N/C ratio, ratio between relative protein levels of vimentin N-term and C-term fragments. Statistical analyses were performed using the ANOVA (Kruskal-Wallis) test.

We performed immunoblots of valve tissue using specific polyclonal antibodies raised against synthetic peptides contained in vimentin N- and C-terminal portions. Fig 1 shows the positions and epitope scores of the chosen peptides. Fig 2 shows results of relative levels of proteins staining with antisera against vimentin N-terminus and C-terminus peptides; it also shows the ratio of relative protein levels of proteins stained with the antisera. The N/C ratio is significantly higher among valve samples from RHD than MXD patients (p = 0.03) (Fig 2).

**Fig 3:**
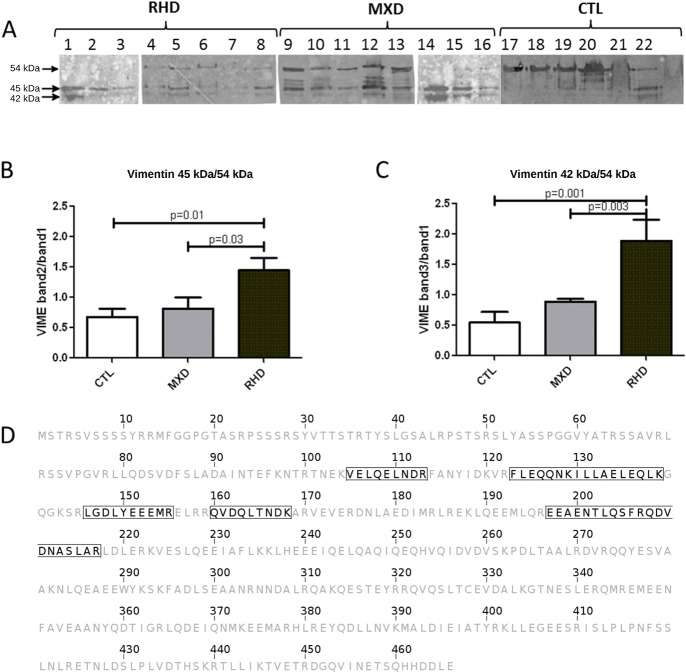
A cleaved form of vimentin was more abundant in RHD valve samples. **(A)** Immunoblots showing vimentin expression in valve samples of RHD (N = 8), MXD (N = 8) and controls (CTL, N = 6). (**B)** and **(C)** Densitometry analysis of the relative expression between band 2 (45 kDa), or band 3 (42 kDa), and 1 (54 kDa). (**D)** Complete sequence of vimentin. Highlighted sequences (boxes) represent peptide sequences of RHD valve spots of 2D-gel analysis previously identified by mass spectrometry (LC-ESI-MS/MS)[7]. Statistical analysis was performed using the ANOVA (Kruskal Wallis) test. *p*-value< 0.05 was considered significant.

Using immunoblotting and densitometry analysis, we characterized the intact form of vimentin with a molecular mass of 54 kDa (Fig 3A, arrow 1). Its expression was detected in control valves and most MXD valves. Vimentin isoforms of 45 kDa and 42 kDa (Fig 3A, arrows 2 and 3, respectively) were mainly detected in valves of RHD patients. A correlation between intact protein and its isoforms was established, and we observed that the expression of the 45-kDa isoform was approximately 2-fold higher in the valves of RHD patients compared with valves of MXD patients (p = 0.03) and the control group (p = 0.01) (Fig 3B). The 42-kDa isoform was the most prevalent isoform in the valve samples of RHD patients compared with both controls and MXD valves (p = 0.001 and p = 0.003, respectively) (Fig 3C). A complete sequence of vimentin is presented in Fig 3D, and the highlighted boxes represent various peptides from the N-terminal portion of the protein previously identified by mass spectrometry that allowed the identification of vimentin isoforms, reinforcing the data obtained above that the vimentin fragment present in RHD valves is cleaved at its C-terminus [7].

### Evaluation of collagen VI, lumican and vitronectin proteins in RHD and MXD valves

**Fig 4:**
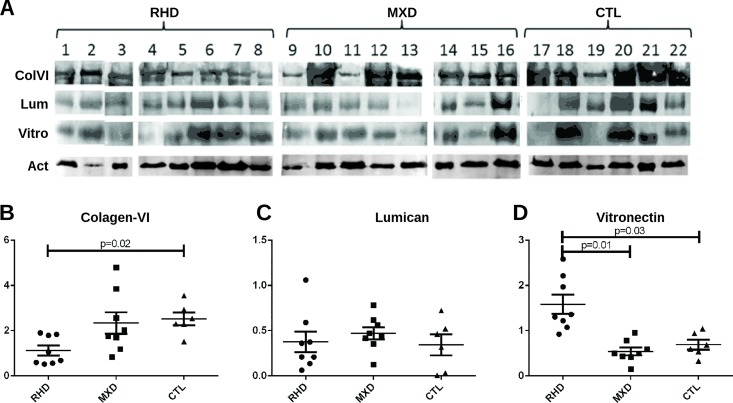
Relative expression of collagen-VI, lumican and vitronectin in the valve tissue of RHD patients. **(A)** Immunoblots showing collagen-VI, lumican and vitronectin expression in RHD (n = 8), MXD (n = 8) and controls (CTL, n = 6) valve samples. (**B, C, D)** Densitometry analyses of collagen-VI alpha-chain, vitronectin and lumican were determined. RHD valve tissue (n = 8), MXD (n = 8) and controls (n = 6). Statistical analysis was performed using the ANOVA (Kruskal-Wallis) test. *p-*value< 0.05 was considered significant.

Collagen VI, lumican and vitronectin proteins were also evaluated by immunoblot (Fig 4A). Altered expression was observed in valves of RHD patients. Reduced collagen VI alpha chain was observed in valve tissue of RHD patients compared with both MXD and control valves (p = 0.02) (Fig 4B), whereas both RHD and MXD patients had similar levels of lumican expression compared with controls (Fig 4C). In contrast, vitronectin, a matrix proteoglycan, displayed increased expression in RHD valves compared with MXD valves (p = 0.01) and control valves (p = 0.03) (Fig 4D).

**Fig 5:**
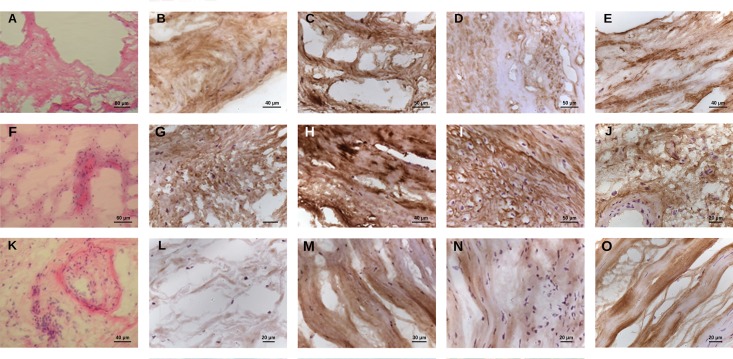
Mitral valve immunohistological pattern expression of vimentin, collagen VI and lumican in damaged valves of RHD and MXD patients. Mitral valve samples of one cadaveric donor (**A)** and MXD (**F**) and RHD (**K**) patients were stained by hematoxylin-eosin (HE). Immunoperoxidase reactions performed for cadaveric donor, MXD patient (#15) and RHD patients with regurgitation (# 6) and stenosis (#5) for vimentin (**B**, **C**, **D** and **E**); collagen VI (**G**, **H**, **I**, **J**) and lumican (**L**, **M**, **N**, **O**).

The immunohistochemical expression of collagen VI, lumican and vimentin in valve tissue was also examined *in situ* in both RHD and MXD valve lesions and was compared with control valves. Collagen VI presented intense and diffuse staining in both RHD and MXD valves. Lumican showed diffuse and intense staining in both MXD and RHD valves with regurgitation and irregular staining in stenotic valves. Vimentin presented diffuse and uniform pattern in MXD although non-uniform in RHD valves (Fig 5).

### Co-localization of the collagen VI alpha chain and lumican

**Fig 6:**
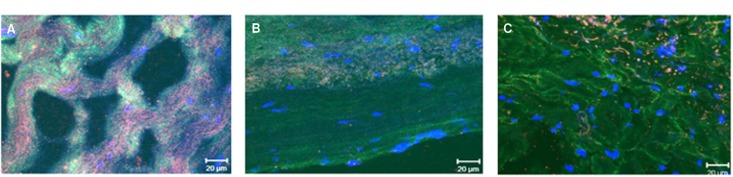
Co-localized expression of collagen VI and lumican in MXD valvular tissue and RHD stenotic valve analyzed by confocal microscopy. (**A**) Control valve tissue from cadaveric transplantation donor; (**B**) Valve tissue from MXD patient (#15); (**C**) Valve tissue from RHD patient with stenosis (# 6). Red- collagen type VI; green- lumican; blue—DAPI.

The co-localization of the collagen VI alpha chain and lumican in RHD and MXD valves revealed a diverse pattern of distribution, in which RHD valves presenting with stenosis displayed an altered distribution of both proteins, with collagen VI on the fibers surrounded by lumican, whereas a diffuse distribution of collagen VI co-localized with lumican, more similar to that observed in control valves, was found in MXD valves (Fig 6).

## Discussion

As RHD and MXD present diverse etiologies and mechanisms that culminate in valve damage, in the present work our aim was to evaluated the pattern of distribution of some proteins in the valve tissue of both diseases. Interestingly, we observed that collagen VI protein presented similar pattern in the valve tissue of both diseases, while other proteins here analyzed displayed distinct pattern.

The process that mediates RHD valvular regurgitation and/or stenosis involves several inflammatory mediators that attract leukocytes, monocytes, T- and B-lymphocytes and soluble mediators, resulting in local inflammation and tissue injury with subsequent cellular matrix disorganization leading to valvular dysfunction.

Myxomatous degeneration also results in valvular dysfunction due to diverse mechanisms that causes mucopolysaccharide accumulation into the valvular matrix, resulting in fragmentation and disruption of the elastic fibers and collagen bundles, leading to valve prolapse and sometimes to chordae rupture [4,5]. This disease is not associated with inflammation, in contrast with RF/RHD, and is the most common valve disease in the United States and Europe [6].

A set of valve proteins with differential and/or altered expression in valvular tissue of both RHD and MXD patients was recently evaluated using a proteomic approach. Among the identified proteins, vimentin, lumican, collagen VI and vitronectin exhibit increased expression exclusively in RHD valves [7]. Vimentin is an important target protein of autoimmune reactions in RHD. In addition, it has been shown that inflammatory cytokines present in rheumatic valve tissue interferon-gamma and TNF-alpha induced changes in expression of matrix proteins in rheumatic valve explants [9].

Previous large-scale proteomic analysis (bidimensional polyacrylamide gel electrophoresis-resolved valve proteins) disclosed a 42 kDa fragment of vimentin as upregulated in RHD valve tissue, while the intact 54 kDa molecule was downregulated in MXD; according to mass spectrometry data, the vimentin fragment appeared to be truncated at the C-terminus [7]. Vimentin is a structural protein that has several functions of note the stabilization of collagen mRNAs [10].

Interestingly, in the present work, an intact form of vimentin (54kDa) and increased amounts of two smaller fragments-possibly proteolytic- (45 and 42 kDa) were identified indicating significant cleavage/degradation of this protein in RHD valves.

The finding of increased N/C terminus protein level ratio and both 42/54 and 45/54 kDa protein level ratios reinforce each other and strongly suggest the fragments come from the N-terminal region of vimentin and that therefore the proteolytic cleavage must have occurred at the C-terminus.

Increased levels of matrix metalloproteinase I were found in the plasma of RHD patients with valve disease [11]; moreover, protein interaction maps generated with Ingenuity Pathways Analysis have previously shown a possible involvement of MMP-25 targeting vimentin, suggesting that increased proteolytic fragmentation could be involved in the generation of smaller molecular weight N-terminal vimentin isoforms. It was also observed a disorganized distribution of this protein in valve tissue samples of RHD patients mainly in those with valve regurgitation, which differs from MXD patients who presented accentuated loss of valvular matrix without alterations in vimentin expression.

The cross-reactive immunological recognition of vimentin was indirectly demonstrated several years ago using monoclonal antibodies obtained from mice immunized with M5 protein[12]. In addition, our group demonstrated that vimentin was one of the targets of auto reactive attack mediated by peripheral T cells and heart tissue-infiltrating T cell clones from several RHD patients, confirming the role of this protein in the pathogenesis of RHD valvular lesions [13].

Although diverse mechanisms are responsible for valve dysfunction in both diseases, it seems that increased interstitial cellularity in RHD, with consequent increased expression of vimentin, can results in structural and functional changes.

Another protein associated with RHD valve tissue injury events is vitronectin, an adhesive glycoprotein that provides a regulatory link between cell adhesion and invasion. This protein binds to some integrins and is important for the maintenance of tissue integrity [14]. The finding that vitronectin shows an increased expression exclusively in the RHD valve tissue, compared with MXD and control valves, implies a prominent role in valve tissue remodeling. It has been shown that vitronectin reduces α5β1 integrin activation and fibronectin fibril extension, altering the extracellular matrix structure [15].

Collagen proteins are essential structural components of connective tissue that are responsible for tissue integrity, repair and growth, and 28 different types exhibiting diverse biological properties have recently been identified in humans (reviewed by Ricard-Blum) [16].

Collagen serves as target for adhesion by microorganisms, including *S*.*pyogenes* in the case of RF and RHD, through collagen-binding proteins. Similarities of streptococcal proteins and collagen have been described; however, no human tissue cross-reactivity was reported [17,18]. Diverse types of collagen (I to IV) bind to different types of M proteins through an octapeptide motif [(A/T/E)XYLXXLN] located in the N-terminal portion of M protein type 3 named PARF (peptide associated with rheumatic fever)[19]. This peptide facilitates the colonization of bacteria to the connective tissue and is necessary to bind to collagen IV, a component of basal membranes that is the target of autoantibodies in several rheumatologic diseases [20]. In RF it was proposed that these interactions likely occur through the M protein-PARF motif by direct binding to the CB3 region of collagen type IV, leading to specific antibodies against valve tissue and resulting in RHD lesions [21].

Interestingly, M1 protein defines one of the most virulent *S*. *pyogenes* strains and does not contain a PARF motif, but binds to collagen VI distributed in the lamina propria underneath the epithelial basement with high affinity, resulting in *S*. *pyogenes* colonization [22]. The emm1 strain is most frequently associated with RF episodes, as reviewed by Steer *et al*.[23].

Reduced collagen VI expression, an extra cellular matrix structural constituent that is present as beaded filaments in RHD valves, was mainly observed in patients exhibiting valve regurgitation [7]. This protein is involved in the integrity of the extracellular matrix together with collagen IV.

Also interesting, lumican a protein responsible for collagen VI fibril assembly, as previously described by Chakravarti *et al*. [24], exhibited normal and more diffuse expression in both RHD and MXD valves. Of note, the *in situ* expression of both collagen-VI and lumican in the present work exhibited a clear and distinctive histological pattern among RHD, MXD and control valves. Collagen-VI and lumican co-localized in RHD valves in fibrotic regions. In contrast, in control valves and MXD patients, collagen-VI was diffuse and localized at the center of the valve fibers, whereas lumican was localized at the periphery. These alterations in lumican/collagen distribution and structure probably interfere with its biological functions, as adhesion, migration and proliferation, by affecting its binding to molecules such as integrins and other structural components [25]. In RHD, integrins play important roles as they are a superfamily of cell adhesion receptors that bind to the extracellular matrix, cell surface and soluble ligands.

In conclusion, distinct patterns of altered valve expression and tissue distribution/organization of structural/matrix proteins have been found in RF/RHD and MXD valvopathy. This may be related to the inflammatory nature of RHD valve lesions. Such differences may play important pathophysiological roles in both diseases.

## Supporting Information

S1 Fig Electrophoretic pattern of mitral valve proteins. 1D polyacrylamide gel shows the pattern of mitral valve proteins from RHD and MXD and control valves.
(TIF)
