# Supplementary figures and images for: Rheumatic Heart Disease and Myxomatous Degeneration: Differences and Similarities of Valve Damage Resulting from Autoimmune Reactions and Matrix Disorganization
Source: PLoS One. 2017 Jan 25;12(1):e0170191. doi: 10.1371/journal.pone.0170191 (PMC5266332; doi:10.1371/journal.pone.0170191)

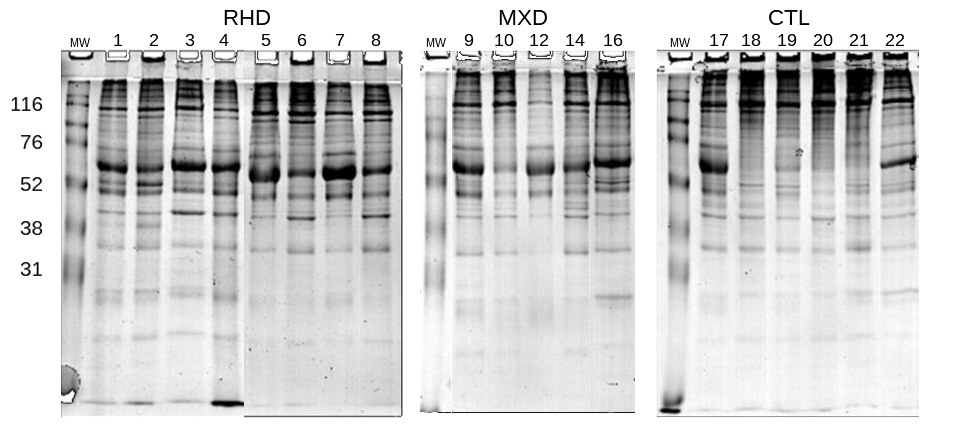

Supplement: S1 Fig — 1D polyacrylamide gel shows the pattern of mitral valve proteins from RHD and MXD and control valves. (TIF) [file pone.0170191.s001.tif]
